# HFIP: an integrated multi-omics data and knowledge platform for the precision medicine of heart failure

**DOI:** 10.1093/database/baab076

**Published:** 2021-11-13

**Authors:** Jing Wu, Min Zhao, Tao Li, Jinxiu Sun, Qi Chen, Chengliang Yin, Zhilong Jia, Chenghui Zhao, Gui Lin, Yuan Ni, Guotong Xie, Jinlong Shi, Kunlun He

**Affiliations:** Research Center of Medical Big Data, Chinese PLA General Hospital, 28 Fuxing Road, Beijing 100853, China; Research Center of Medical Big Data, Chinese PLA General Hospital, 28 Fuxing Road, Beijing 100853, China; Research Center of Medical Big Data, Chinese PLA General Hospital, 28 Fuxing Road, Beijing 100853, China; Research Center of Medical Big Data, Chinese PLA General Hospital, 28 Fuxing Road, Beijing 100853, China; Research Center of Medical Big Data, Chinese PLA General Hospital, 28 Fuxing Road, Beijing 100853, China; Research Center of Medical Big Data, Chinese PLA General Hospital, 28 Fuxing Road, Beijing 100853, China; Research Center of Artificial Intelligence, Chinese PLA General Hospital, 28 Fuxing Road, Beijing 100853, China; Research Center of Biomedical Engineering, Chinese PLA General Hospital, 28 Fuxing Road, Beijing 100853, China; Ping An Healthcare Technology, 316-1 Laoshan Road, Beijing 200120, China; Ping An Healthcare Technology, 316-1 Laoshan Road, Beijing 200120, China; Ping An Healthcare Technology, 316-1 Laoshan Road, Beijing 200120, China; Ping An Healthcare and Technology Co, Ltd, 316-1 Laoshan Road, Shanghai 200120, China; Ping An International Smart City Technology Co, Ltd, 5033 Yitian Road, Shenzhen 518046, China; Research Center of Medical Big Data, Chinese PLA General Hospital, 28 Fuxing Road, Beijing 100853, China; Research Center of Medical Big Data, Chinese PLA General Hospital, 28 Fuxing Road, Beijing 100853, China

## Abstract

As the terminal clinical phenotype of almost all types of cardiovascular diseases, heart failure (HF) is a complex and heterogeneous syndrome leading to considerable morbidity and mortality. Existing HF-related omics studies mainly focus on case/control comparisons, small cohorts of special subtypes, etc., and a large amount of multi-omics data and knowledge have been generated. However, it is difficult for researchers to obtain biological and clinical insights from these scattered data and knowledge. In this paper, we built the Heart Failure Integrated Platform (HFIP) for data exploration, fusion analysis and visualization by collecting and curating existing multi-omics data and knowledge from various public sources and also provided an auto-updating mechanism for future integration. The developed HFIP contained 253 datasets (7842 samples), multiple analysis flow, and 14 independent tools. In addition, based on the integration of existing databases and literature, a knowledge base for HF was constructed with a scoring system for evaluating the relationship between molecular signals and HF. The knowledge base includes 1956 genes and annotation information. The literature mining module was developed to assist the researcher to overview the hotspots and contexts in basic and clinical research. HFIP can be used as a data-driven and knowledge-guided platform for the basic and clinical research of HF.

**Database URL**: http://heartfailure.medical-bigdata.com

## Introduction

Heart failure (HF), the terminal phenotype of many cardiovascular diseases, is a complex and heterogeneous syndrome (1). It is a growing public health problem, leading to considerable morbidity and mortality (2). Due to the extensive burden of the disease coupled with the complexity of HF syndrome, the signs and symptoms are often deceptive and the suspected patients cannot be fully diagnosed (3). With the development of bioinformatics technology, many researchers have started to assist in the diagnosis of HF by studying the molecular mechanisms and looking for biomarkers of HF. Currently, the genetic and epigenetic mechanisms of HF have been extensively studied using multi-omics data and genome-wide association analysis to demonstrate the genetic variations and transcriptional comparisons, reveal the differential expression and epigenetic analysis, and show the potential modification mechanisms.

The high-throughput sequencing technology provides a new idea for the diagnosis and treatment of HF. There has been a rapid accumulation of effective omics data and knowledge. Discovering pathological mechanisms and mining knowledge from these data is an effective way for basic and clinical researchers. However, the proliferation and independent dissemination of data have brought significant challenges for researchers in data analysis to obtain meaningful insights. Organizing and analyzing these data with HF as a unit can provide a convenient way for researchers to quickly acquire effective datasets and knowledge. The discovery of data mining will also provide an important basis for revealing disease pathogenesis and clinical treatment.

Currently, some databases for gene–disease associations and data collection are relatively comprehensive, but do not focus on a specific disease. ClinVar is a public database that collects genetic variants related to diseases. It integrates information on four aspects, i.e. variation, clinical phenotype, empirical data and functional annotation (4, 5). DisGeNET provides information on gene–disease associations, variant–disease associations and disease–disease associations by integrating data from expert-curated repositories, genome-wide association (GWAS) catalogs, animal models and scientific literature (6, 7). Online Mendelian Inheritance in Man (OMIM) focuses on the relationships between human genetic variation and phenotypic traits (8). However, it is very time-consuming to query and screen from these databases to obtain genetic information about HF. Furthermore, the information on these datasets is scattered in Gene Expression Omnibus (GEO) (9), Sequence Read Archive (SRA) (10) and other databases, and the relevant knowledge is also diamond-shaped in various knowledge bases and literature. For clinical and scientific workers, it is very difficult to retrieve and analyze data about HF without separate centralized reflection. Therefore, a comprehensive data platform that contains datasets, knowledge and tools for HF is necessary.

To fill this gap, we focused on HF and attempted to construct an integrated platform consisting of multi-omics data, easy-to-use tools and relevant molecular knowledge, namely the Heart Failure Integrated Platform (HFIP), by automatically collecting and manually organizing relevant datasets and knowledge, and performing intelligent matching analysis and visualization tools on selected datasets. This platform is a valuable resource for researchers and clinicians to conduct studies and practice in HF.

## Methods and results

**Figure 1.**
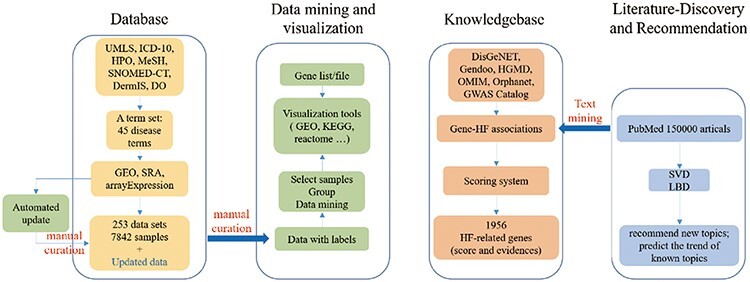
The construction framework of HFIP.

In order to build a comprehensive HF omics database, we acquired HF-related omics datasets and genomic events from existing databases and text mining and performed data mining and visualization with corresponding tools (Figure 1). HFIP mainly includes five basic function modules: ‘Database’, ‘data automatic update’, ‘Tools’, ‘Knowledgebase’, and ‘Literaturediscovery’.

Focusing on HF, we systematically interpreted a given disease name into a full set of disease terms (Supplementary file1). Then, various types of omics datasets were collected based on this term set to form a specialized disease database. In addition, an automatics collection tool was used to update the newly released datasets.

Gene- and dataset-oriented analysis and visualization tools were also provided separately. The former was designed to reveal the gene variants, expression and regulatory activities in different datasets, and the latter was developed to compare different disease progression states. Both of them provide a flexible and easy-to-use web approach for public and user-own data, which is important for basic and clinical researchers who are not familiar with bioinformatics tools. Based on these systematically collected datasets, new molecular events could be identified.

To construct a complete knowledge base of HF-related genetic events, gene–HF associations were recognized from all types of public databases and literature. All these associations were integrated to form a complete disease-omics knowledge graph which could be used for precision reasoning and decision for the diagnosis and treatment of HF.

It is important to find a good research idea. Thus, a literature discovery module was also designed to represent the research hotspots related to HF in this platform. The knowledge about gene–HF associations extracted from this literature was also put into the ‘Knowledgebase’ to make the information about HF-related genes more abundant. Finally, an interaction platform was established to facilitate direct data mining and knowledge retrieval.

## Data collection and curation

### Data collection

The first step in data and knowledge collection, sharing, and exchange is to construct the standardizing disease term set of HF. Considering lexical heterogeneity of HF, we integrated the possible names from several sources: (i) UMLS, Unified Medical Language System (11), (ii) ICD-10, International classification of diseases-version 10, (iii) HPO, human-phenotype-ontology (12), (iv) MeSH, Medical Subject Headings, (v) SNOMED-CT, Systematized Nomenclature of Medicine-Clinical Terms, (vi) Medscape, (vii) DermIS, Dermatology Online Atlas, and (viii) DO, Human Disease Ontology (13). Finally, a complete list of 45 disease terms was obtained (Supplementary file1).

**Figure 2.**
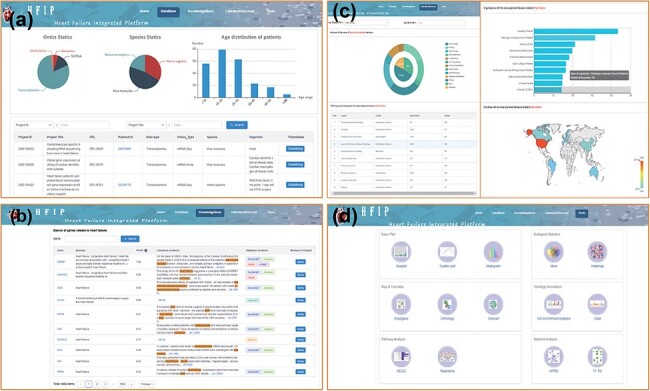
Four-function modules of HFIP. (a) Database; (b) Knowledge base; (c) Literature Base and (d) Tool pool.

Using the term set of HF as keywords, we collected HF-related datasets from the three main repositories for multi-omics data, i.e. GEO, SRA and ArrayExpress (14). After manual calibration and curation, 253 datasets and about 7842 samples, including three omics, i.e. genome, transcriptome and methylation (with the proportions of 5.00%, 92.08% and 2.92%, respectively), and three species, i.e. *Homo sapiens*, *Rattus norvegicus* and *Mus musculus* (with the proportions of 33.18%, 19.90% and 46.92%, respectively), were obtained to summarize the existing omics studies of HF (Figure 2a).

### Data mining and visualization

**Figure 3.**
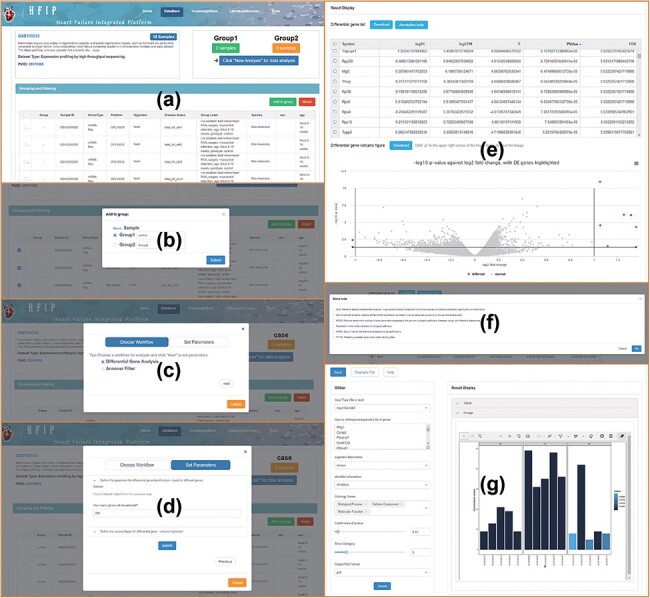
The process of data mining, including data screening, grouping, analysis and visualization in HFIP.

Through carefully manual calibration, labels of disease progression, sample status, organism and project descriptions have been added to each sample. Based on these labels, users can screen, group and perform secondary data mining in a single dataset. Gene-oriented and dataset-oriented search and analysis were provided. Some tools of multi-omics data analysis were designed and integrated for all these datasets, including differential expression analysis, variation annotations, network module detection, etc. Corresponding visualizations were also provided, which can be used to reveal the internal biological insight straightforwardly. Different tools can be intelligently filtered and matched to each dataset of different omics characteristics. Take the dataset of ‘GSE100532’ as an example, the data mining process is as follows (Figure 3): (i) clicking ‘DataMining’ to start data analysis, (ii) clicking ‘Add to group’ to group samples, (iii) clicking ‘Click New Analysis for data analysis’ to select data analysis process, (iv) setting the parameters, including differential expression analysis and Annotate Variation (ANNOVAR) tool (15), (v) generating data analysis results, such as differentially expressed genes, volcano maps, etc., (vi) accessing the gene list function display and so on, such as enrichment and reactome, and (vii) displaying the result of Gene Ontology (GO) pathway enrichment of differentially expressed genes. These related workflows were built on the galaxy system (https://galaxyproject.org/) to implement scheduling management.

**Figure 4.**
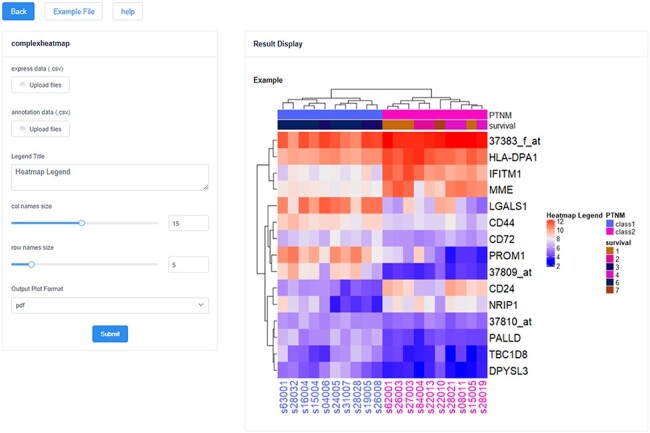
The heatmap visualization tool in HFIP. The left side is the data upload and parameter adjustment panel, and the right side is the result display and export panel.

In addition, these analysis and visualization tools formed a tool pool, including 14 tools (Figure 2d) (i)—Basic Plot: Boxplot, Scatter plot and Histogram (ii); Biological Statistics: Venn and Heatmap (iii); Map and Translate: Snp2gene, Orthology and Convert (iv); Ontology Annotation: GO enrichment analysis and Gost (16); (v) Pathway Analysis: Kyoto Encyclopedia of Genes and Genomes (KEGG) and Reactome (17); and (vi) Network Analysis: Human Protein Reference Database (HPRD) (18) and analysis of transcription factor regulatory network (TF-TG). It can provide not only multiple-dimensional analysis and visualization for the datasets in the ‘Database’ but also a separate application entry. Users can directly fill in or import the gene list of their concern into the tool for analysis and achieve visualization shows, and the results can be downloaded in pdf, png and jpg formats (Figure 4). These tools all support the applications of multiple gene types and multiple species.

### Automatic data update and curation

In order to achieve continuous accumulation of data, an automatic updating module was implemented by resolving the structural omics data records in the main public database. According to the determined 45 HF items, an automatic extraction program was designed for GEO and SRA databases. We used the R package ‘GEOmetadb’ (19), ‘GEOquery’ (20) and ‘SRAdb’ (21) to periodically obtain the description of the latest datasets and samples and download the selected data. As of 31 October 2019, the system had automatically extracted 1206 datasets and 13 765 samples.

In order to ensure the accuracy of the datasets related to HF, a review mechanism was established. All automatically updated data were stored on the MongoDB database in the form of metadata. The administrator can review and manage the data through the data update management page, including adding labels to each sample. Based on the metadata description information or the literature information, two labels, i.e. ‘Disease Status’ and ‘Group Label’, will be manually added to each sample, and other labels can be obtained through text mining. After manual review, the data can be released. They were downloaded, processed and finally merged into the database.

## Knowledge collection

In order to facilitate clinicians or researchers to quickly obtain HF-related genes, we systematically integrated gene–HF associations from OMIM, ClinVar, DisGeNET and other databases, as well as information from literature mining based on confirmed HF keywords. At present, the knowledge base already contains 1956 HF-related genes and their corresponding mutation sites. Each gene–HF association is supported by evidences, including publications, representative sentences describing the association, and the HFIP score (Figure 2b). The HFIP score was computed using a scoring system based on Phenolyzer’s scoring model and knowledge automatically from literature (22). The score range is 0 to 1 and concrete rules are as follows:

- Data collection: We first obtained genetic disease datasets from DisGeNET (6), Gendoo (23), Human Gene Mutation Database (HGMD) (24), OMIM (25), Orphanet (26) and GWAS Catalog (27).
- Data screening: The standardizing HF term set was matched with the gene–disease association data to obtain the gene–HF associations.
- Extraction of gene–HF associations from literature: Based on text mining and machine learning methods, we have discovered 4069 unique relationships among diseases and genes, drugs, tests and surgery from approximately 150 000 articles related to HF. The sentences describing gene–HF associations in the articles were displayed in the knowledge base as supporting evidence, and the impact factors of the corresponding articles were also saved.
- Construction of weighted model: Due to the differences in gene–disease data obtained from different databases and articles published in journals of different quality, we established a weighted model in order to get a comprehensive score. The different databases and the description of the gene–HF associations in a single database were given different scores according to the reliability of its expression. The scores of gene–HF associations in DisGeNET and Gendoo were extracted. As for HGMD, it is professional knowledge base information that has been manually verified, so its score is set to 1. Others come from the scores of OMIM, GWAS Catalog and Orphanet after normalization in Phenolyzer. The weight ratio between the knowledge bases was HGMD:DisGeNET:Gendoo:OMIM:GWAS Catalog:Orphanet = 2:1.5:1.5:1:1:1. The impact factors and the number of publications were also added to the weighted module as quantitative indicators. The impact factor ranges correspond to the score of 0–1: 0.1, 1–2: 0.2, 2–3: 0.3, 3–4: 0.4, 4–6: 0.5, 6–8: 0.6, 8–10: 0.7, 10–15: 0.8, 15–20: 0.9 and >20: 1. The weight of knowledge base and literature mining was set to 0.6:0.4.
- The score of each gene was finally normalized to the range of 0–1. The weighted model satisfies the following relationship (22):

(1) \begin{align*} S& \left( {Gene,Term} \right) \nonumber\\ &\!\!\!\!\!\!\!\!\!\!=\! {{\mathop \sum \nolimits_{Diseas{e_i}\ in\ Disease} Score\left( {Gene,Diseas{e_i}} \right) \!\times\! Reliability\left( {Diseas{e_i}} \right)} \over {Count\left( {Disease} \right)}}\end{align*}

where $S\left( {Gene,Term} \right)$ is the weighted score of the gene–term association. $Term$ represents one of the terms extended by HF (Supplementary file2), such as cardiac failure and congestive heart failure. $Diseas{e_i}$ includes the diseases or phenotypes related to the term. $i$ is the serial number of the disease or phenotypes. $Score\left( {Gene,Diseas{e_i}} \right)$ comprises the corresponding scores between the *i*-th disease or phenotype related to the term and a gene. ${\rm{Reliability}}\left( {Diseas{e_i}} \right)$ is the reliability of the *i*-th disease. $Count\left( {Disease} \right)$ is the number of diseases or phenotypes related to the term.

The normalized model is as follows (22):

(2) \begin{equation*}\tilde s\left( {Gene,Term} \right) = {{S\left( {Gene,Term} \right)} \over {max\left\{ {S\left( {Gene,Term} \right)} \right\}}}\end{equation*}

Where $max\left\{ {S\left( {Gene,Term} \right)} \right\}$ represents the maximum value of the correlation score between the gene and the term.

A higher score indicates a stronger degree of association. Researchers can use this as a reference to quickly check the contribution of the candidate genes to HF, thereby narrowing the range of candidate genes. In order to facilitate users to query and judge the reliability of the gene–HF association, we set up a gene search window. The basic information, HF-related mutation sites of the gene and a network diagram of gene–HF associations can be obtained from the window.

## Literature discovery and recommendation

Researchers rely on knowledge to generate new assumptions, especially in the domain of medicine. In order to automatically develop new hypotheses and predict the prevalence of existing topics, literature-based discovery algorithms were applied to a large number of published articles. Based on the key HF items, we systematically collected related knowledge items from existing databases including OMIM, ClinVar, DisGeNET, Gendoo, HGMD, Orphanet, Genome-Wide Association Studies database (GWASdb), Leiden Open Variation Database (LOVD), Pharmacogenomics Knowledgebase (PharmGKB), The Genotype-Tissue Expression (GTEx) and genome database (genomeDB) in the form of a triple of <SUB, REL, OBJ>, where SUB was HF-related items, OBJ was the types of related entities such as gene, drug, lab tests, etc., and REL was the relationship between HF and the object entity. In this article, we have collected all HF-related articles from PubMed (around 150 000 papers). Two types of analysis were conducted to predict the future hot topics: (i) Singular value decomposition method was leveraged to recommend brand new topics in the future. (ii) Time-series-based algorithm was applied to predict the trend of known topics (Supplementary file2). The former was designed to develop new topics in the future, the latter was to predict the prevalence of a given research topic. All these results constituted the ‘LiteratureBase’.

**Figure 5.**
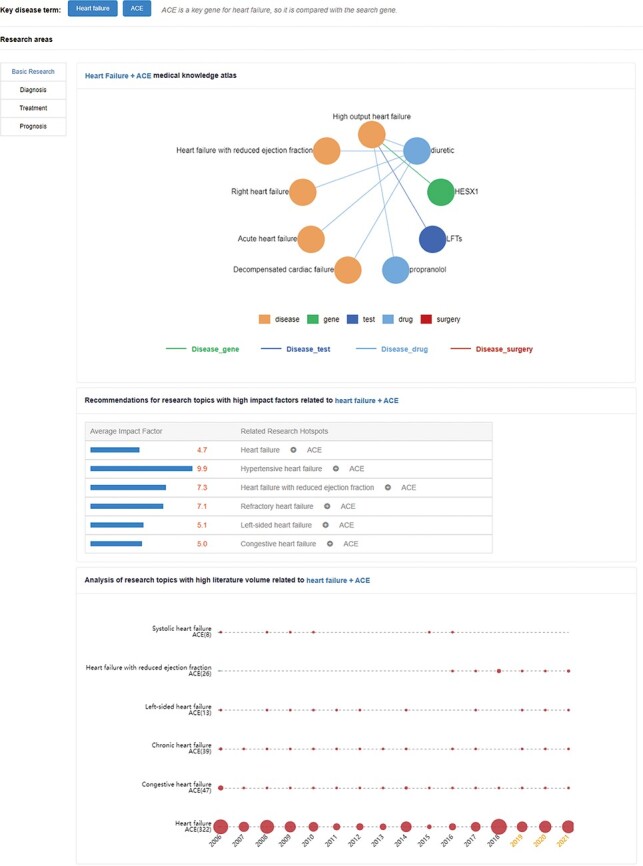
Research hotspots and future research trends of an angiotensin-converting enzyme (ACE) gene in HF. The upper network diagram is the medical knowledge map. The middle part is recommendations for high-impact-factor research topics related to HF + ACE. These numbers indicate the average impact factor of related literature. The lower part is the research topic analysis, and the area of the circle represents the heat of the relation.

The ‘LiteratureBase’ shows the field of HF-related analysis, journals, organizations and countries with more reports on HF (Figure 2c). Users can enter the types of HF and genes in the search window to view the hot development trend of the gene in different fields of HF and the hottest genes currently studied in this field (Figure 5).

## Discussion

With the explosive growth of omics data, we have shifted from data accumulation to data analysis. These data applications greatly rely on data mining and knowledge collection. However, they are widely distributed in different locations in different forms. Thus, integrating and managing these data and knowledge is the first step. In order to build an integrated platform with HF as a theme, we collected a lot of HF-related datasets and gene–HF associations, embedded many analysis and visualization tools, and finally constructed a user-friendly web interface. This is crucial for the systematic investigation of HF pathologies or molecular mechanisms.

As a comprehensive platform for HF research, the HFIP provides enriched HF-related datasets, 1956 HF-related genes, HF-related research hotspots and 14 visualization tools. Each dataset in HFIP includes data description information such as GEO ID, omics type, species, organism, disease status, and gene expression level and mutations. These data labels and tools used in HFIP allow greater flexibility in performing data analysis and visualization. The developed platform is very convenient and effective for scientific research and clinical workers working on HF.

## Future work

To provide new HF-related datasets, we will continuously update the datasets through the modules of automatic updating and manual verification in HFIP. The gene–HF associations from text mining will also be continuously added to the knowledge base, and the specific role of genes on HF will be more clarified. This platform will help medical research to gain more knowledge and assist clinical decision-making through the increased data and knowledge accumulated in HFIP. The HFIP should also greatly contribute to a better understanding of underlying mechanisms for complex HF disease.

## Supplementary Material

baab076_Supp
