# Supplementary material for: HFIP: an integrated multi-omics data and knowledge platform for the precision medicine of heart failure
Source: Database (Oxford). 2021 Nov 13;2021:baab076. doi: 10.1093/database/baab076 (PMC8607296; doi:10.1093/database/baab076)
Supplement: baab076_Supp [file baab076_supp.zip › Supplementaryfile2.doc]

**Supplementary materials**

**LBD methods**

Researchers rely on knowledge to generate new assumptions, especially in the domain of medicine. To automatically develop new hypotheses and predict prevalence of existing topics, literature-based discovery algorithms are applied to large number of published articles. A topic is defined as a tripe as <SUB, REL, OBJ> where SUB is heart failure, OBJ is the kinds of entity that are related such as gene, drug, lab tests and etc. and REL is the relationship between the heart failure with the object entity. In this article, we have collected all heart failure related articles from PubMed (around 150,000 papers) and conducted two kinds of analysis to predict the future hot topics:

- Singular value decomposition(SVD) method is leveraged to recommend brand new topics in the future
- Time series based algorithm is applied to predict the trend of known topics.

**SVD method**

Focusing on heart failure, we extract a couple of relationships by cooccurrence from PubMed articles to generate training and testing set. From about 150,000 heart failure related articles, this generate 4069 unique relationships of diseases and genes, drugs, tests and surgeries. We define relationships which are published before 2014 as training set and those after 2014 as testing set. The SVD methods translate training set to a binary adjacent matrix, in which columns represents disease and rows represents genes, drugs, tests and surgeries. If a relationship occurs in the training set, the value will be set to 1, otherwise the value is 0. By decomposition and reconstruction of the matrix by several singular value, each value in the matrix is approximately set between 0 and 1, which is the score of each relationship. We sort the value and give the top-N recommendation. This method get 40% precision in top-10, and 20% precision of brand new topics, which means it occurs for the first time in the future. Some other results are shown in Table S1.

Table S1 top-N precision of SVD methods

| Top-N | 5 | 10 | 20 | 50 | 100 |
| --- | --- | --- | --- | --- | --- |
| precision | 20% | 40% | 40% | 36% | 32% |
| precision* | 0% | 20% | 20% | 16% | 11% |

*represents precision of brand new topics

**Time series prediction**

SVD method focuses on develop new topics in the future, however it cannot predict the prevalence of such topics, which is also important to researchers. We count the published number of each relationship from 1979 to 2018. The number of first 35 years is treated as training data and we use the published number in last 5 years to evaluate the performance of time series prediction methods. The result shows that RMSE of the last 5 years is 1.334. An example is shown in Figure S1.

**Figure S1.** An example of time series prediction.

In our recommendation system, the most 6 popular relations of a specific disease will be shown to the researchers, including the future 3 year’s trend we predict. An example of congestive heart failure trend prediction is shown in Figure S2. The area of the circle represents heat of the relation.

**Figure S2.** The trend prediction of congestive heart failure
